# Sirt5 Deacylation Activities Show Differential Sensitivities to Nicotinamide Inhibition

**DOI:** 10.1371/journal.pone.0045098

**Published:** 2012-09-19

**Authors:** Frank Fischer, Melanie Gertz, Benjamin Suenkel, Mahadevan Lakshminarasimhan, Mike Schutkowski, Clemens Steegborn

**Affiliations:** 1 Department of Biochemistry and Research Center for Bio-Macromolecules, University of Bayreuth, Bayreuth, Germany; 2 Department of Enzymology, Institute for Biochemistry and Biotechnology, Martin Luther University Halle-Wittenberg, Halle, Germany; Semmelweis University, Hungary

## Abstract

Sirtuins are protein deacylases regulating metabolism and aging processes, and the seven human isoforms are considered attractive therapeutic targets. Sirtuins transfer acyl groups from lysine sidechains to ADP-ribose, formed from the cosubstrate NAD^+^ by release of nicotinamide, which in turn is assumed to be a general Sirtuin inhibitor. Studies on Sirtuin regulation have been hampered, however, by shortcomings of available assays. Here, we describe a mass spectrometry–based, quantitative deacylation assay not requiring any substrate labeling. Using this assay, we show that the deacetylation activity of human Sirt5 features an unusual insensitivity to nicotinamide inhibition. In contrast, we find similar values for Sirt5 and Sirt3 for the intrinsic NAD^+^ affinity as well as the apparent NAD^+^ affinity in presence of peptide. Structure comparison and mutagenesis identify an Arg neighboring to the Sirt5 nicotinamide binding pocket as a mediator of nicotinamide resistance, and statistical sequence analyses along with testing further Sirtuins reveal a network of coevolved residues likely defining a nicotinamide-insensitive Sirtuin deacetylase family. The same Arg was recently reported to render Sirt5 a preferential desuccinylase, and we find that this Sirt5 activity is highly sensitive to nicotinamide inhibition. Analysis of Sirt5 structures and activity data suggest that an Arg/succinate interaction is the molecular basis of the differential nicotinamide sensitivities of the two Sirt5 activities. Our results thus indicate a Sirtuin subfamily with nicotinamide-insensitive deacetylase activity and suggest that the molecular features determining nicotinamide sensitivity overlap with those dominating deacylation specificity, possibly suggesting that other subfamily members might also prefer other acylations than acetylations.

## Introduction

Sirtuins are protein deacetylases that hydrolyze one NAD^+^ cosubstrate for each lysine sidechain they deacetylate, which links their activity to cellular energy levels [1], [2]. They have been implicated in lifespan extending effects of caloric restriction and contribute to regulation of stress resistance, metabolism, and aging processes [1], [3]. These physiological roles have stimulated intensive research into functions and substrates of Sirtuins, their physiological regulation, and small molecule drugs modulating their activity [3], [4], [5], [6]. Mammals have seven isoforms, Sirt1 to Sirt7, with diverse functions in the nucleus (Sirt1, Sirt6, Sirt7), cytosol (Sirt2), and mitochondria (Sirt3, Sirt4, Sirt5) [7]. Mitochondrial Sirt3 regulates a large number of metabolic enzymes [8], [9], [10], [11] and shows the typical apparent Sirtuin affinity for the cosubstrate NAD^+^ (K_M_ in the range 0.1–0.6 mM) and sensitivity for product inhibition (K_i_≤200 µM) by nicotinamide [12], [13]. In contrast, little is known about substrates and regulation of another mitochondrial isoform, Sirt5. No kinetic data for nicotinamide and NAD^+^ are available due to low Sirt5 deacetylase activity in available assays, and only one physiological substrate is known, carbamoyl phosphate synthetase 1 (CPS1). Sirt5-dependent deacetylation activates CPS1 and the urea cycle and increases during fasting, indicating that Sirt5 might contribute to caloric restriction effects [14], [15]. Sirt5 was recently found to show higher lysine desuccinylation and demalonylation activity *in vitro* compared to its low deacetylation activity and it can desuccinylate CPS1 *in vivo* [16]. Studying the sofar unknown physiological functions of these acyl modifications and their Sirt5-dependent removal should help to improve our understanding of Sirt5 function. Sirt5 was also suggested to contribute to malignant diseases [17], but its pathophysiological role, targets, and regulation largely remain to be revealed [14].

Sirtuins share a common catalytic core topology, but with variations e.g. in the linker between Rossman fold and Zn^2+^-binding domain [18]. The substrate polypeptide chain binds between these domains, next to NAD^+^. The acetyl oxygen reacts with C1′ of NAD^+^, yielding an ADP-ribosyl peptidyl imidate and nicotinamide. The reaction can then proceed to deacetylated lysine and 2′-O-acetyl-ADP-ribose, or, after rebinding of nicotinamide, reversal of the first step leads to substrate reformation and apparent inhibition of deacetylation [19], [20]. NAD^+^ and nicotinamide are assumed to be general physiological Sirtuin regulators [21], but little is known about their regulatory effects on less studied Sirtuins, including several mammalian isoforms.

Studies on Sirtuin regulation have been hampered by shortcomings of the available substrates and activity assays [22], [23]. The widely used “Fluor de Lys” (FdL) assay [24] and many other optical assays rely on peptides with a chemical label, which restricts them to a limited number of synthetic substrates and can cause artefacts such as the fluorophor-dependent activation of Sirt1 in the FdL assay [25], [26]. Optical assays in general, including those employing non-modified substrates such as ELISAs [10], HPLC/UV [27], and coupled enzymatic assays [23], suffer from sensitivity to optical interference, e.g. from metabolites or pharmacological modulators. HPLC/UV assays thus require complete isolation of the quantified product [27], which often requires optimization for individual targets and prevents their application to complex mixtures, such as protein digests or lysates. Coupled assays are further sensitive to interferences with downstream reactions, and antibody-based methods are often not site specific. Many of these shortcomings can be overcome by using mass spectrometry-based assays, which were used, for example, in peptide experiments identifying consensus substrate sequences and for profiling endogenous deacetylase activities [28]. Furthermore, mass spectrometry is increasingly used for studying acetylations of whole proteins, in particular in proteomics studies (see e.g. [29], [30]). In principal, this approach enables label-free analysis, but stable isotope labeling is normally used for relative quantification [30], [31].

Here, we describe a robust mass spectrometry deacylation assay based on direct and label-free relative quantification of substrate and product. Using the assay, we show that human Sirt5 deacetylase activity exhibits an unusual insensitivity for inhibition by physiological nicotinamide concentrations. The enzyme's desuccinylation activity, in contrast, is potently inhibited. Structure comparisons and mutagenesis reveal molecular features responsible for these effects, and evolutionary analyses suggest the existence of a Sirtuin family with a nicotinamide insensitive deacetylase activity.

## Results and Discussion

### A label-free quantitative deacylation assay using mass spectrometry

**Figure 1:**
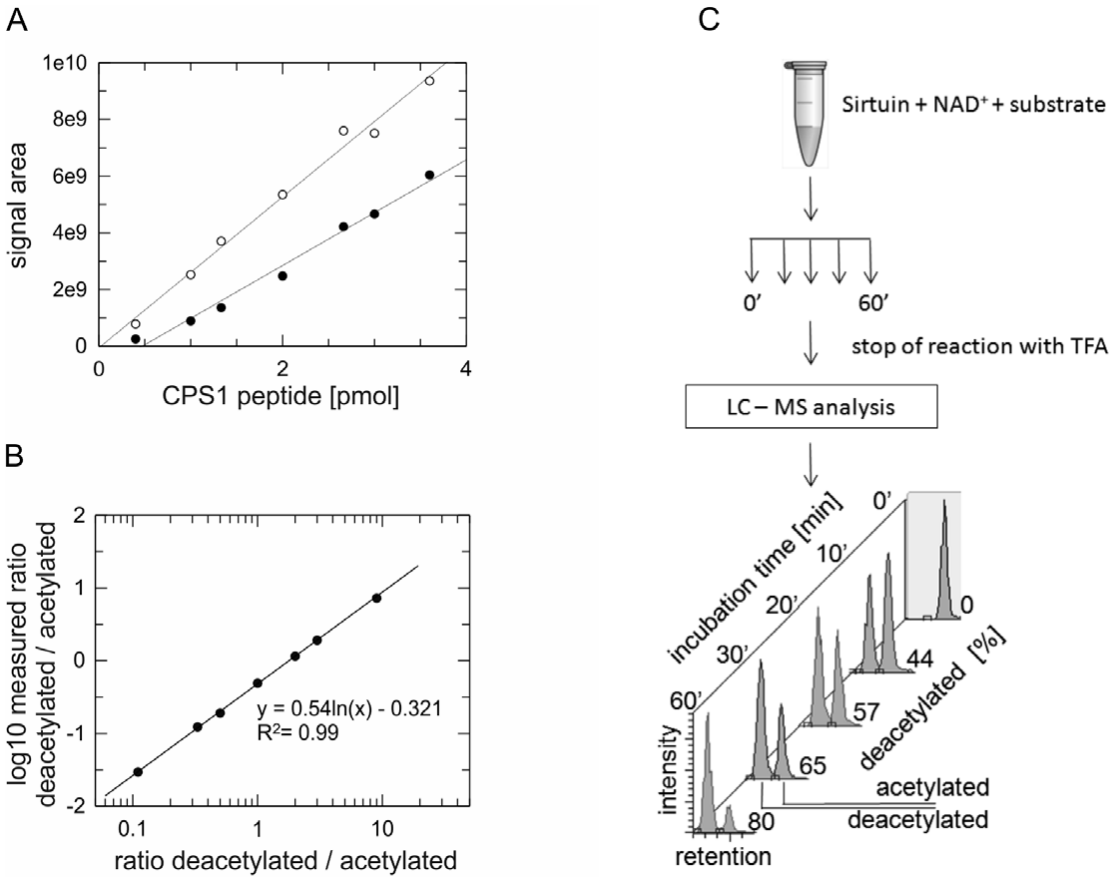
Development of a label-free, quantitative mass spectrometry-based deacylation assay. (**A**) Different amounts of acetylated (○) and deacetylated (•) CPS1-Lys527 peptide are plotted against their respective mass spectrometry signal areas. Interpolations (lines) show the linear correlations between peptide amounts and detected signals, and the slightly different slopes for the two peptide species. (**B**) Ratios of the injected amounts of deacetylated and acetylated peptide plotted against ratios of the measured log_10_ signal areas (•). Equation and correlation for the linear interpolation (line) are indicated. (**C**) Scheme for the mass spectrometry-based deacylation assay. Percent deacetylation is calculated by normalizing the product area to the total signal area, and deacetylation rates are determined through analysis of aliquots taken after different incubation times.

Sirt5 tests in our lab with the FdL deacetylation assay seemed to indicate an unusual insensitivity to nicotinamide inhibition, but data reliability was limited due to weak signals since the acetylated FdL-1 peptide is a bad Sirt5 substrate. Through peptide array experiments (unpublished data) we identified CPS1-Lys527 (FKRGVL(acK)EYGVKV) as a candidate for a better, and likely physiological, Sirt5 substrate site and we confirmed this result using an enzyme-coupled assay and binding studies (Fig. S1A). However, since the enzyme-coupled assay is based on nicotinamide consumption it can not be applied for studying Sirtuin inhibition by nicotinamide. To study effects of this compound and other substances not compatible with optical assays, we therefore established a robust and quantitative, mass spectrometry-based assay. Simultaneous measurement of substrate and product would enable the necessary normalization for run-to-run comparability, but that requires binding of both peptide species, acetylated and deacetylated, to the chromatography column. Surprisingly, several acetylated and deacetylated peptides tested did not bind to a C18 reversed phase column using standard LC-MS conditions, e.g. 0.1% formic acid, as loading buffer. A major influence of buffer and counter ions on peptide retention is well known, and we therefore tested more hydrophobic ones to increase in particular retention of the more hydrophilic deacetylated peptides. Using 0.1% TFA and 0.02% HFBA turned out to be suitable for all tested peptides (data not shown). For accurate measurements of relative changes in acetylation without labeling, we then aimed at the parallel quantification of product and substrate. The novel Sirt5 substrate peptide CPS1-Lys527 was used for this systematic analysis. Calibration curves for acetylated and deacetylated peptide revealed that substrate and product can be monitored accurately (Fig. 1A). Even though a slight but systematic under determination of the deacetylated peptide occurs, normalization of the product with the remaining substrate allows relative quantification of acetylated and deacetylated peptide and increases run-to-run comparability (Fig. 1B). The principle of our deacylation assay is schematically shown in Fig. 1C for the Sirtuin-dependent deacetylation of a histone H3 acetylation site (H3-Lys116, IHK(acK)RVT) as an example. The simple and robust assay is attractive for *in vitro* testing of compounds due to its insensitivity to many compound features disturbing other assays, and it can be applied for the monitoring of specific deacetylation sites in substrates from synthesized peptides to whole proteins, even in complex mixtures.

### Human Sirt5 deacetylase activity shows an unusual insensitivity to nicotinamide inhibition

**Figure 2:**
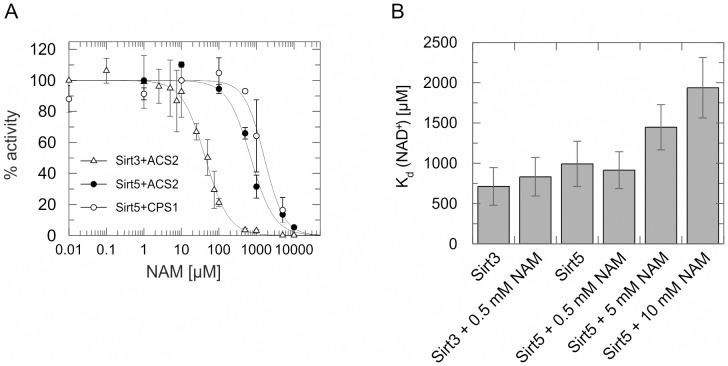
The mass spectrometry-based deacetylation assay reveals an unusual low Sirt5 sensitivity for nicotinamide inhibition. (**A**) Dose-dependent nicotinamide inhibition of the deacetylation of an ACS2 or CPS1 peptide, respectively, by Sirt3 and Sirt5. % activity was determined through relative quantification of reaction product by mass spectrometry and normalization to the respective non inhibited activity (set to 100%). Error bars represent standard errors for three independent measurements. (**B**) Dissociation constants for the interaction of NAD^+^ with Sirt3 and Sirt5, respectively, in presence of different nicotinamide concentrations. K_d_ values were determined by microscale thermophoresis measurements. Error bars represent standard errors of three independent measurements. NAM, nicotinamide.

We then used our mass spectrometry assay to analyze the effects of nicotinamide on Sirt5, and on other Sirtuins for comparison. To ensure that different deacetylation activities, e.g. due to different efficiencies against different substrates or the presence of inhibitors, resulted in endpoint signals within the optimal measurement range, we adjusted reaction times and enzyme amounts using initial time series experiments. Applying the assay to the deacetylation of a peptide based on an acetyl-CoA synthetase 2 acetylation site (ACS2-Lys642, TRSG(acK)VMR) by human Sirt3 in presence of increasing nicotinamide concentrations showed inhibition in the low micromolar range (IC_50_ = 43±3 µM; Fig. 2A), similar to previous results on this and several other Sirtuins [12], [13]. For deacetylation of CPS1-Lys527 peptide by human Sirt5, we observed a much lower sensitivity to nicotinamide inhibition (IC_50_ = 1.6±0.3 mM; Fig. 2A). Sirt5 thus is fully active in presence of up to ∼100 µM nicotinamide, which is assumed to correspond to the higher end of cellular concentrations [21], whereas a major inhibitory effect requires non-physiological concentrations.

For other Sirtuins, a form of nicotinamide insensitivity was caused by a high ratio k_forward_ to k_reverse_, leading to fast turnover of the ADP-ribosyl-peptidyl imidate and thereby preventing the nicotinamide-initiated reverse reaction of the intermediate [19]. In these cases, an incomplete loss of activity was observed at nicotinamide levels of 50 to 100 µM, and no further increase in inhibition at higher concentrations. No such initial drop in activity was observed in the Sirt5/nicotinamide dose-response. However, to test whether the substrate and its specific turnover rate influence the behavior of Sirt5, we analyzed inhibition of Sirt5 activity against ACS2-Lys642, which is deacetylated slower by Sirt5 than CPS1-Lys527 (ACS2: 1.6±0.1 nmol mg^−1^ min^−1^, CPS1: 4.1±0.1 nmol mg^−1^ min^−1^; Fig. S1A). The Sirt5 affinity for CPS1 and ACS2 substrates was comparable (CPS1: K_d_ = 2.3±1.5 µM, ACS2: K_d_ = 2.1±0.7 µM; Fig. S1B), and also comparable to Sirt3's affinity for the ACS2-Lys642 peptide (K_d_ = 2.4±1.0 µM; Fig. S1B), showing that the difference in deacetylation is due to different turnover. Nicotinamide inhibited Sirt5-dependent ACS2-Lys642 deacetylation with an IC_50_ of 0.7±0.1 mM (Fig. 2A), about 2-fold stronger than deacetylation of CPS1-Lys527. This result shows that nicotinamide inhibition of Sirt5 exhibits weak substrate dependence, but Sirt5 inhibition for both substrates is similar, compared to the order of magnitude stronger inhibition observed for the Sirt3/ACS2-Lys642 reaction. In fact, turnover by Sirt5 should be faster for physiological substrates than for the likely non-physiological ACS2-Lys642 site, so that in case of a negative correlation between turnover and nicotinamide sensitivity an even weaker inhibition than for ACS2-Lys642 would be expected. We thus conclude that nicotinamide inhibition of Sirt5-dependent deacetylation features weak substrate-dependence but appears to generally require high, non-physiological nicotinamide concentrations.

### Sirt5 has a typical Sirtuin NAD^+^ affinity and is inhibited competitively by nicotinamide

Nicotinamide is the leaving group of the Sirtuin cosubstrate NAD^+^. The low nicotinamide sensitivity of Sirt5 indicates that compared to other Sirtuins, differences in the nicotinamide subpocket of the NAD^+^ binding site exist, which might also result in a lower NAD^+^ affinity. However, determining NAD^+^ affinities of Sirt3 and Sirt5 revealed an only slightly lower affinity for Sirt5 (Sirt3 K_d_(NAD^+^) = 0.71±0.23 mM; Sirt5 K_d_(NAD^+^) = 0.98±0.28 mM; Fig. 2B, Fig. S2A), not correlating with the order of magnitude difference in nicotinamide sensitivity. Similarly, K_d_ values for NAD^+^ under peptide saturation were comparable for Sirt3 (0.26±0.15 mM) and Sirt5 (0.20±0.07 mM; Fig. S2B). Thus, differences between Sirt5 and other Sirtuins in the NAD^+^ binding site appear not to affect pocket features responsible for cosubstrate affinity but might be restricted to features determining details of nicotinamide rebinding (see below).

Nicotinamide-sensitive Sirtuins were shown to be inhibited non-competitively with respect to NAD^+^ through a base exchange mechanism [19]. Consistently, NAD^+^ affinities of Sirt3 and Sirt5 are not altered in presence of 0.5 mM nicotinamide (Sirt3 K_d_(NAD^+^) = 0.83±0.24 mM; Sirt5 K_d_(NAD^+^) = 0.92±0.23 mM; Fig. 2B, Fig. S2A), a concentration not affecting Sirt5 but completely inhibiting Sirt3. However, addition of increased nicotinamide concentrations that affect Sirt5 activity (5 mM and 10 mM, respectively) resulted in increased binding constants for NAD^+^ to Sirt5 (K_d_(NAD^+^) = 1.44±0.28 mM at 5 mM nicotinamide, K_d_(NAD^+^) = 1.94±0.37 mM at 10 mM nicotinamide; Fig. 2B, Fig. S2A). Consistent with our findings, kinetic studies showed competitive binding of nicotinamide and NAD^+^ to Sirtuins at nicotinamide concentrations above 2 mM [19]. We thus conclude that the surprising insensitivity of Sirt5 to physiological nicotinamide concentrations is due to a lack of non-competitive inhibition via base exchange, and that the inhibition observed at very high nicotinamide concentrations is likely caused by competition with NAD^+^.

### Molecular features contributing to the nicotinamide resistance of Sirt5

**Figure 3:**
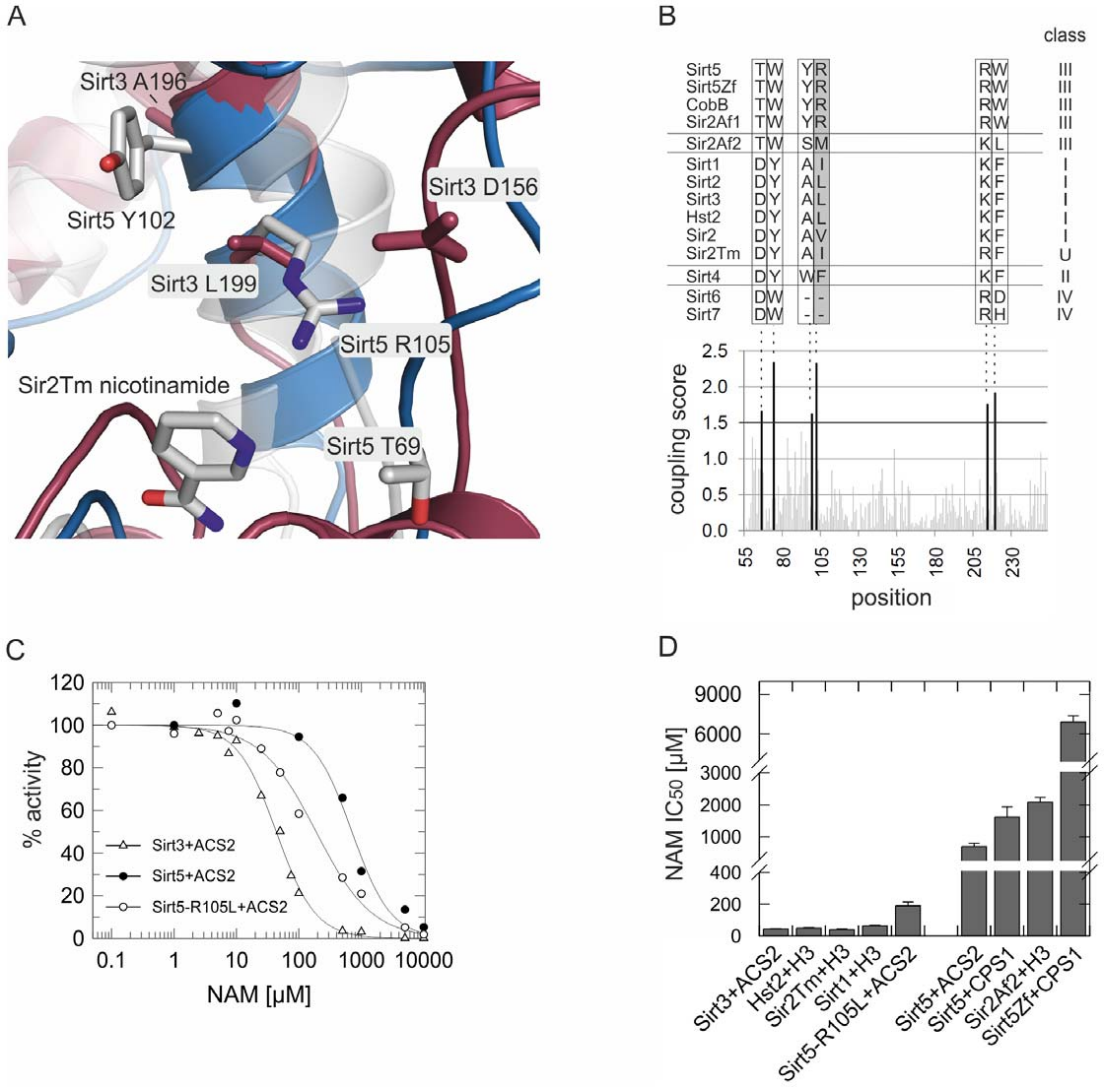
Structure comparison and identification of a Sirtuin sequence motive indicating nicotinamide insensitive deacetylation activity. (**A**) Structure comparison of Sirt3 (red) and Sirt5 (blue), overlaid with a Sir2Tm/nicotinamide complex (grey). Nicotinamide and coupled residues (see below) in Sirt3 and Sirt5 are displayed as sticks and colored by atom type for Sirt5 and for nicotinamide. (**B**) Statistical coupling analysis scores (lower panel) identify residues apparently coevolving (score cutoff used: 1.5) with Sirt5-Arg105: Thr69 and Tyr102 (highlighted in panel A), Trp77, Arg217, and Trp222. Amino acids in other Sirtuin sequences and the corresponding Sirtuin class are shown on top of the scores. (**C**) Inhibition Sirt5-Arg105Leu by nicotinamide. Activities, determined using our mass spectrometry assay, were normalized against activity in absence of nicotinamide. Data for wildtype Sirt3 and Sirt5 from Fig. 2A are shown here for comparison. (**D**) Comparison of IC_50_ values, determined using the mass spectrometry assay, for nicotinamide inhibition of class I and III Sirtuins and of the Sirt5-Arg105Leu variant. Error bars represent the standard error of the fit. NAM, nicotinamide.

To identify molecular Sirt5 features responsible for its insensitivity to physiological nicotinamide levels, we compared its structure to other Sirtuins. For visualization of nicotinamide in the so-called C-pocket of the NAD^+^ binding cleft, we used the structure of a *Thermotoga maritima* Sir2 (Sir2Tm)/nicotinamide complex [20]. Overlaying this structure with human Sirt3 [32] and Sirt5 [33] revealed that the C-pockets are extremely similar but as a major difference close to the C-site we found Arg-105, which corresponds to a Leu in the nicotinamide sensitive Sirt3 (Fig. 3A). Arg-105 points toward the C-pocket and might interfere with the nicotinamide binding orientation required for the inhibitory base-exchange reaction. To identify additional candidate residues for rendering a nicotinamide binding site inhibition sensitive or insensitive, we searched for residues coevolved with Arg105 through statistical coupling analysis [34] of 932 aligned Sirtuin sequences. The amino acids in the five positions showing highest coupling to Arg105 (coupling scores >1.5; Fig. 3B) form a network of residues located within the nicotinamide binding site (Thr69, Tyr102; Fig. 3A) or adjacent to it (Trp77, Arg217, Trp222). To test the roles of these residues in nicotinamide sensitivity, we generated Sirt5 variants carrying at positions 105 and 69, respectively, the corresponding Sirt3 amino acid (Sirt5-Arg105Leu and Sirt5-Thr69Asp). Exchanging Thr69, which is located in the so called flexible cofactor binding loop, abolished Sirt5 activity. A Sirt5-Arg105Leu variant, however, retained wildtype deacetylation activity (wt: 2.1±0.4 nmol mg^−1^ min^−1^; Arg105Leu: 2.4±0.1 nmol mg^−1^ min^−1^) and partially gained Sirt3-like nicotinamide sensitivity (IC_50_ = 189±23 µM; Fig. 3C). Thus, Arg105 appears to play a major role in rendering Sirt5 nicotinamide-insensitive, possibly assisted by coevolved residues.

Among the five phylogenetic Sirtuin classes [35], Sirt5 belongs to class III, which is considered evolutionary “old” and contains mostly bacterial members. Even though Arg105 and the coupled residues are not strictly conserved within this class, their variations match well with this classification (Fig. 3B). Consistently, we found nicotinamide sensitivities comparable to Sirt3 for the class I and class U members human Sirt1 (IC_50_ = 62±4 µM), yeast Hst2 (IC_50_ = 49±2 µM), and Sir2Tm (IC_50_ = 39±5 µM; Fig. 3D, Fig. S3). In contrast, the class III Sirt5 ortholog from zebrafish (Sirt5Zf), which features the identical amino acids as human Sirt5 in the Arg105-centered network, did not respond to physiological nicotinamide concentrations (IC_50_ = 6.9±0.5 mM; Fig. 3D, Fig. S3). Furthermore, the bacterial class III member Sir2 from *Archaeoglobus fulgidus* (Sir2Af2), which has an only slightly different residue pattern in the Arg105 network, also showed nicotinamide insensitivity (IC_50_ = 2.1±0.15 mM; Fig. 3D, Fig. S3). In particular, the central Arg is replaced in Sir2Af2 by Met, which apparently also interferes with proper nicotinamide binding through its long sidechain (see below), showing that variations in the Arg105-centered motif occur in nicotinamide-insensitive Sirtuins. Although many members of the mainly prokaryotic class III Sirtuins would need to be tested for a firm conclusion, we speculate that besides a large group of nicotinamide sensitive Sirtuins, there might exist a family of Sirt5-like nicotinamide-insensitive Sirtuins, which likely corresponds to class III or a subfamily thereof.

### The nicotinamide-sensitivity of Sirt5 depends on the type of acyl group

**Figure 4:**
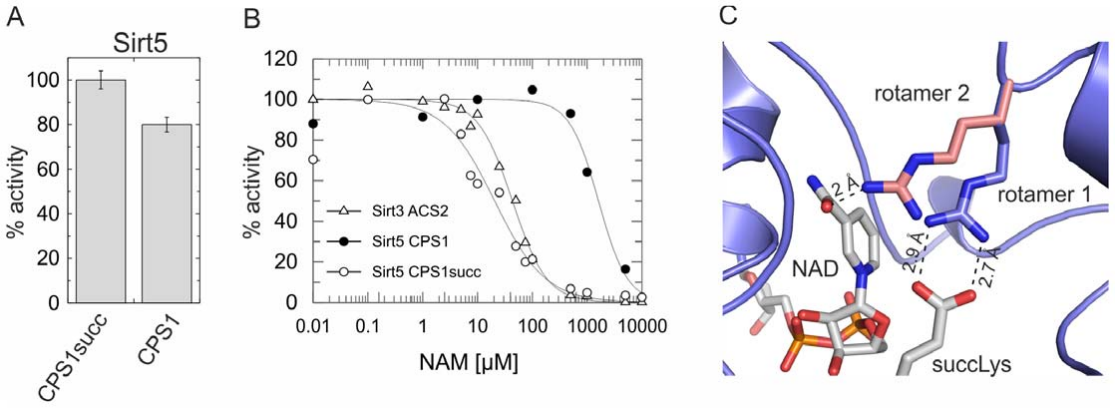
Nicotinamide effects on Sirt5 activity depend on the type of acyl group. (**A**) Comparison of Sirt5 activities against acetylated and succinylated CPS1-Lys527 peptide, determinded by mass spectrometry and normalized using the desuccinylation activity. Error bars represent standard errors of linear fits to time-series experiments. (**B**) Nicotinamide-dependent inhibition of Sirt5 activity against acetylated and succinylated CPS1-Lys527 peptide, determined using mass spectrometry and normalized against activity in absence of nicotinamide. The Sirt3/ACS2 and Sirt5/CPS1 curves from Fig. 2A were added for comparison. (**C**) Cartoon representation of the Sirt5/succinylated substrate/NAD^+^ complex structure. The ligands and Arg105 are shown as sticks colored according to atom type (ligands) or in blue (Arg105). A modeled alternative sidechain conformation for Arg105 (rotamer 2) is shown in red. CPS1succ, succinylated CPS1-Lys527 peptide; NAM, nicotinamide.

Recent publications showed an increased deacylation activity of Sirt5 for succinylated rather than acetylated Lys, at least partly due to lower K_M_ values [16], [36]. Interestingly, Du *et al.* identified similar residues as our study, Arg105 and the coupled residue Tyr102, to be important for the desuccinylase activity [16]. We therefore tested a succinylated version of the CPS1 peptide (CPS1-Lys527succ, benzoyl-RGVL(succK)EYGV-amide) as Sirt5 substrate in our mass spectrometry assay, and analyzed the sensitivity of the reaction to nicotinamide inhibition. Consistent with published data [16], Sirt5 deacylated the succinylated peptide more efficiently than the acetylated one (Fig. 4A), although the difference was small in our assays due to the high substrate concentrations used (500 µM). Surprisingly, the desuccinylation reaction showed a nicotinamide sensitivity (IC_50_ = 21±4 µM; Fig. 4B) comparable to class I Sirtuins, revealing that Sirt5 deacetylation and desuccinylation activities are differently regulated by nicotinamide.

**Figure 5:**
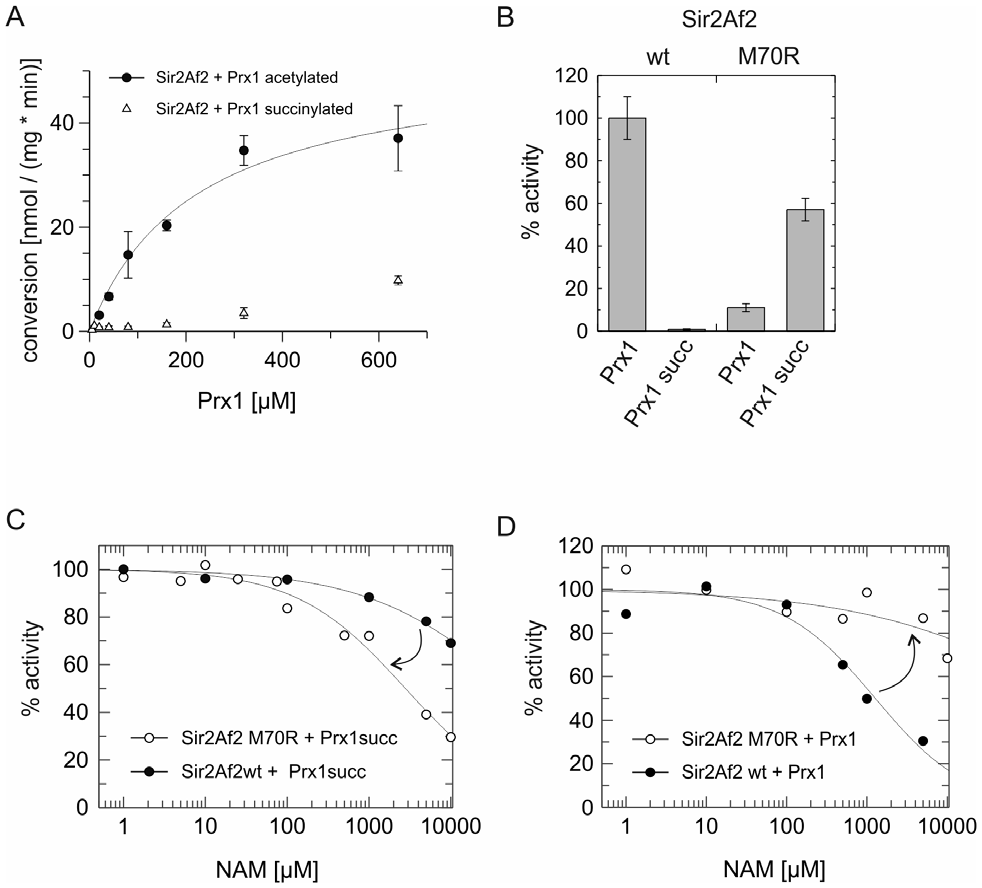
Role of Sir2Af2-Met70 in deacylation specificity and nicotinamide sensitivity. (**A**) Substrate concentration dependent activity of Sir2Af2 against acetylated (•) and succinylated (Δ) Prx1-Lys197 peptides determined using the mass spectrometry assay. Error bars represent standard errors of linear fits to time-series measurements. (**B**) Comparison of Sir2Af2 activities against acetylated and succinylated Prx1-Lys197 peptide, determined using mass spectrometry and normalized against wt deacetylation activity. Error bars represent standard errors of linear fits to time-series experiments. (**C+D**) Nicotinamide-dependent inhibition of Sir2Af2 wt (•) and Met70Arg variant (○) activity against (C) succinylated and (D) acetylated Prx1-Lys197 peptide, determined using mass spectrometry and normalized against activity in absence of nicotinamide. The effect of exchanging Met70 to Arg is indicated by arrows. Prx1succ, succinylated Prx1-Lys197 peptide; NAM, nicotinamide.

To investigate the molecular basis of the modification-dependent nicotinamide effect, we analyzed the structure of the available Sirt5/succinylated substrate/NAD^+^ complex [16]. As described [16], Arg105 contributes to substrate binding through an interaction with the succinyl carboxylate (Fig. 4C, rotamer 1). Without this interaction, the long Arg sidechain is likely to sample other conformations, as Arg adopts 33 conformations in proteins with comparable frequency [37]. Several Arg rotamers possible in absence of the succinate interaction, i.e. with acetylated substrates, point toward the nicotinamide binding pocket (see Fig. 4C, rotamer 2 for an example) and thus should interfere with proper nicotinamide binding for inhibition. To test this hypothesis, we analyzed deacylation reactions and nicotinamide effects using Sir2Af2, which has a Met replacing Arg105 (Met70). The Met cannot form a salt bridge to succinate - a Sirt5 Arg105Met variant indeed lost the desuccinylase activity [16] - but its long sidechain can point into the nicotinamide pocket. As expected, Sir2Af2 showed insignificant desuccinylation activity (K_M_ and v_max_ not determinable) but pronounced deacetylase activity (K_M_ = 220±56 µM, v_max_ = 52±6 nmol mg^−1^ min^−1^; k_cat_/K_M_ = 132 s^−1^ M^−1^; Fig. 5A and Fig. S4A). Consistent with our hypothesis, the enzyme was insensitive to nicotinamide inhibition independent of substrate sequence (CPS1-Lys527 or Prx1-Lys197, SKEYFS(acylK)QK) and modification being removed (acetyl-Prx1 IC_50_ = 1.3±0.3 mM, succinyl-Prx1 IC_50_>10 mM; acetyl-CPS1 IC_50_ = 5.8±1.6 mM, succinyl-CPS1 IC_50_ = 6.6±0.5 mM, Fig. S4B,C). The increase in IC_50_ for the succinyl versus acetyl modification observed for the Prx1 peptide might indicate that the longer succinyl chain could also contribute to interfering with nicotinamide binding if not fixed in an interaction with an Arg. We then changed Sir2Af2-Met70 to Arg, resulting in the expected increase in desuccinylase and decrease in deacetylase activity (Fig. 5B). Consistent with the model that decreased conformational freedom in a Sir2Af2-Met70Arg/succinyl-peptide complex facilitates inhibitory nicotinamide binding, the nicotinamide sensitivity of the mutant's desuccinylase activity was increased compared to wildtype (IC_50_ changed from >10 mM to 2.8±0.9 mM; Fig. 5C). For acetylated substrates, where the Sir2Af2-Met70Arg variation introduces no ordering interaction but an even larger side chain potentially interfering with nicotinamide binding, we indeed find a further decrease in nicotinamide sensitivity compared to wildtype (IC_50_ changed from 1.3±0.3 mM to >10 mM; Fig. 5D). These results support our hypothesis that this residue (Sirt5-Arg105 respective Sir2Af2-Met70) not only contributes to acyl modification specificity, but that its conformational freedom also influences nicotinamide sensitivity. The fact that the single Sir2Af2 mutation neither fully swapped substrate specificity nor shifted nicotinamide sensitivity completely to class I Sirtuin levels indicates that additional residues contribute to these enzyme properties, consistent with our Sirt5 mutagenesis results (see above).

### Conclusions and outlook

Our mass spectrometry-based assay allows robust and label-free monitoring of deacylation reactions, such as deacetylation and desuccinylation. The obtained activity data suggest that a Sirtuin subfamily exists whose deacetylation activity shows no sensitivity to inhibition by physiological nicotinamide concentrations, and that the conformational freedom of the residue corresponding to Arg105 in human Sirt5 has a major influence on nicotinamide sensitivity. In Sirt5, this residue is conformationally restrained when interacting with the enzyme's preferred substrate acylation, a malonyl or succinyl group, likely causing the increased sensitivity of the Sirt5 desuccinylation activity to nicotinamide inhibition. At present, one cannot exclude the possibility that both activities, deacetylation and desuccinylation, contribute to Sirt5 function, since the more efficient *in vitro* activity desuccinylation can be lowered *in vivo* by nicotinamide, and no quantitative information is available whether acetylated or succinylated Sirt5 substrate sites dominate *in vivo*. However, it is tempting to speculate that a low nicotinamide deacylation sensitivity generally indicates that the identified residue lacks its optimal acyl interaction partner and thus, that a side-activity rather than the major deacylation activity is measured. If this speculation turns out to be true, it will be interesting to see which acylations constitute the physiological substrates for Sir2Af2 and the Sirtuin subfamily whose deacetylation activity shows low nicotinamide sensitivity.

Sirtuins are attractive drug targets [3], [4], [5] and many modulators have been identified through screening methods or rational approaches [4], [6], [38], but most compounds show low potency and/or specificity and no suitable compounds are available for Sirt5. The unique Sirt5 deacylation activity [16] and nicotinamide sensitivity pattern (this work) now indicate a promising approach for drug development: Exploiting the isoform-specific features around the nicotinamide binding pocket investigated here should allow the development of small molecule inhibitors selective for Sirt5 [39], which would be leads for pharmacological drug development and excellent tools for functional studies on Sirt5.

## Methods

### Chemicals

Chemicals were obtained from Sigma (Saint Louis, USA) if not stated differently. Acetylated peptides and succinylated Prx1-Lys197 peptide were from GL Biochem (Shanghai, PRC). Succinylated CPS1 peptide was synthesized as benzoylated amide using rink amide MBHA resin and standard Fmoc solid phase synthesis (20% piperidine in DMF for deprotection and PyBOP/DIPEA for couplings). After treatment with 96% TFA, the peptide was precipitated with diethylether, purified by C18 HPLC, and succinylated using succinic anhydride in DMF in presence of DIPEA, followed by NaOH treatment to remove tyrosine succinylations. Succinylated peptide was purified by C18 HPLC, and purity and identity analyzed using LC-ESI-MS.

### Recombinant expression and purification of human Sirt1, Sirt3, and Sirt5, zebrafish Sirt5, yeast Hst2, A. fulgidus Sir2Af2, and T. maritima Sir2Tm

Proteins were expressed and purified using standard methods described in detail in Methods S1. Briefly, gene fragments were cloned into pET15b (Sirt1; Novagen, Darmstadt, Germany) or pET151/D-Topo (Invitrogen, Carlsbad, USA) and expressed in *E. coli* BL21DE3 (Hst2) or *E. coli* BL21DE3Rosetta2 (all other proteins). Proteins were purified using Talon (Clontech, Mountain View, USA) affinity chromatography followed by gelfiltration on a Superdex 200 (Sirt1) or Superose12 column (all other proteins) (GE Healthcare, Waukesha, USA). Sirt1 was further purified by HiTrapQHP (GE Healthcare) ion exchange chromatography. Expression constructs for Sir2Af2 and Sir2Tm [20], [40] were provided by the Wolberger lab via Addgene (Cambridge, USA) and proteins produced according to [41].

### Coupled deacetylation assay

Coupled deacetylation assays were performed as described [23]. Briefly, reaction mixtures were supplemented with 10 µM Sirt5, 0.5 mM peptide, and 2.5 mM NAD^+^ and the decrease in absorbance at 340 nm was monitored over 1 h at room temperature. Controls were done without Sirt5 and all assays were repeated at least twice (results shown are representatives).

### MS-based peptide deacylation assay

Reaction mixtures contained 20 mM Tris/HCl pH 7.8, 150 mM NaCl, 2.5 mM NAD^+^, 0.5 mM substrate peptide, and varying concentrations of nicotinamide and Sirtuin protein if not stated differently. Deacylations were started by adding peptide and stopped with TFA (0.25% final concentration) after 10 to 60 min incubation at 37°C. Samples were diluted with 0.1% formic acid and filtered using 10 kDa cutoff concentrators. Samples were analyzed on an LC-ESI-MS system consisting of a Prominence HPLC (Shimadzu, Duisburg, Germany) with Sil20AC autosampler connected to an LTQ XL mass spectrometer (Thermo Fisher, Bremen, Germany). 1 pmol peptide was injected and species separated using a linear gradient from 0% to 45% buffer B within 30 min (buffer A: 0.1% TFA, 0.02% HFBA; buffer B: 70% ACN, 0.1% TFA, 0.02% HFBA) and a fritless 100 µm ID capillary reversed phase column (Reprosil C18 AQ, 3 µm; Dr. Maisch, Germany) with a flow rate of 250 nl/min. Full MS scans between 375 and 1600 m/z, followed by full MS/MS scans for the three most intensive ions were acquired using Xcalibur 2.1 software. Extracted ion chromatograms (XICs) with mass windows of ±2 m/z were generated for the acylated and deacylated peptide, and peak areas were determined using automatic peak area detection of the Xcalibur Qual Browser module and further analyzed with Excel (Microsoft, Seattle, USA) or GraFit (Erithacus Software, West Sussex, UK). MS raw data for all figures are assembled in Table S1.

### Binding analysis by microscale thermophoresis

For labeling, 100 µM Sirtuin were incubated with 200 µM fluorescein isothiocyanate (Thermo Fisher) in 20 mM HEPES pH 7.5, 150 mM NaCl over night at 4°C in the dark. Non-bound dye was removed using a Nap25 column (GE Healthcare). Dissociation constants were determined from microscale thermophoresis [42] using a Monolith NT.115 (NanoTemper Technologies, Munich, Germany). Ligand was titrated to 100 nM labeled Sirtuin in presence or absence of nicotinamide (0.5/5/10 mM) or substrate peptide (25 µM), mixtures preincubated for 30 min at room temperature, and thermophoresis measured with an excitation wavelength of 470 nm, emission wavelength 520 nm, LED-power 20–50%, and laser-power 40%. Dissociation constants were determined by non-linear fitting in GraFit.

### Statistical coupling analysis

Covariation of residues was calculated using the method and software of Lockless *et al.* [34] and an alignment of human Sirt5 with 932 Sirtuin sequences of the PFAM database (http://www.sanger.ac.uk/resources/databases/pfam.html) manually edited in Genedoc [43].

### Structure analysis

The alternative rotamer of Arg105 in the Sirt5/succinylated peptide/NAD^+^ complex (PDB ID 3RIY, [16]) and the structure overlay of human Sirt3 (3GLS [32]), human Sirt5 (2NYR [33]), and *T. maritima* Sir2Tm (1YC5 [20]) were generated using Coot [44]. Structure images were prepared using PyMol (Delano Sci. LLC, http://www.pymol.org).

## Supporting Information

Figure S1 **Improved Sirt5 substrates and their binding to Sirt5.**
(PDF)

Figure S2 **Microscale thermophoresis experiments for determination of dissociation constants for NAD^+^ binding to human Sirt3 and Sirt5.**
(PDF)

Figure S3 **Nicotinamide (NAM) dose response curves for various Sirtuins.**
(PDF)

Figure S4 (PDF)

Methods S1 (PDF)

Table S1 **Mass spectrometry raw data for Figures 1–5.**
(XLSX)
