# Supplementary material for: Sirt5 Deacylation Activities Show Differential Sensitivities to Nicotinamide Inhibition
Source: PLoS One. 2012 Sep 19;7(9):e45098. doi: 10.1371/journal.pone.0045098 (PMC3446968; doi:10.1371/journal.pone.0045098)

Suppl. Fig. S1 Improved Sirt5 substrates and their binding to Sirt5. (A) Continuous assays for Sirt5-dependent deacetylation of ACS2-Lys642 and CPS1-Lys527. Linear interpolations are shown as lines. Data are representatives of two replicates. (B) Microscale thermophoresis analysis of binding of acetylated CPS1 and ACS2 peptide, respectively, to Sirt3 and Sirt5, through titration with increasing peptide amounts. Error bars represent standard errors for three independent measurements .

A

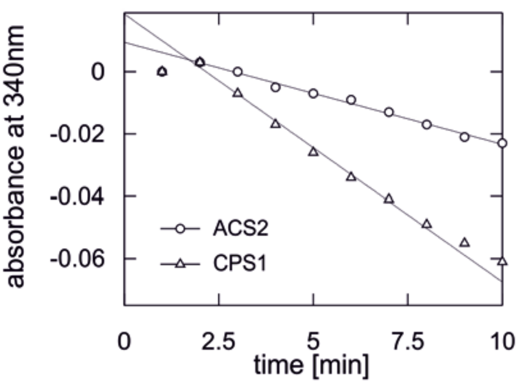

B

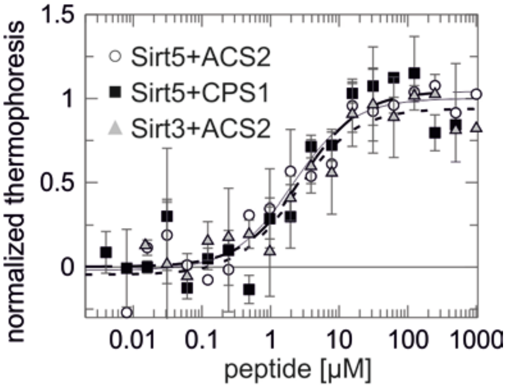

Supplement: Figure S1 — Improved Sirt5 substrates and their binding to Sirt5. (PDF) [file pone.0045098.s001.pdf]
