# Supplementary material for: Sirt5 Deacylation Activities Show Differential Sensitivities to Nicotinamide Inhibition
Source: PLoS One. 2012 Sep 19;7(9):e45098. doi: 10.1371/journal.pone.0045098 (PMC3446968; doi:10.1371/journal.pone.0045098)
Supplement: Figure S2 — Microscale thermophoresis experiments for determination of dissociation constants for NAD+ binding to human Sirt3 and Sirt5. (PDF) [file pone.0045098.s002.pdf]

Suppl. Fig. S2: Microscale thermophoresis experiments for determination of dissociation constants for NAD⁺ binding to human Sirt3 and Sirt5 (A) in absence or presence of nicotinamide (NAM) or (B) in absence or presence of 25 µM substrate peptide

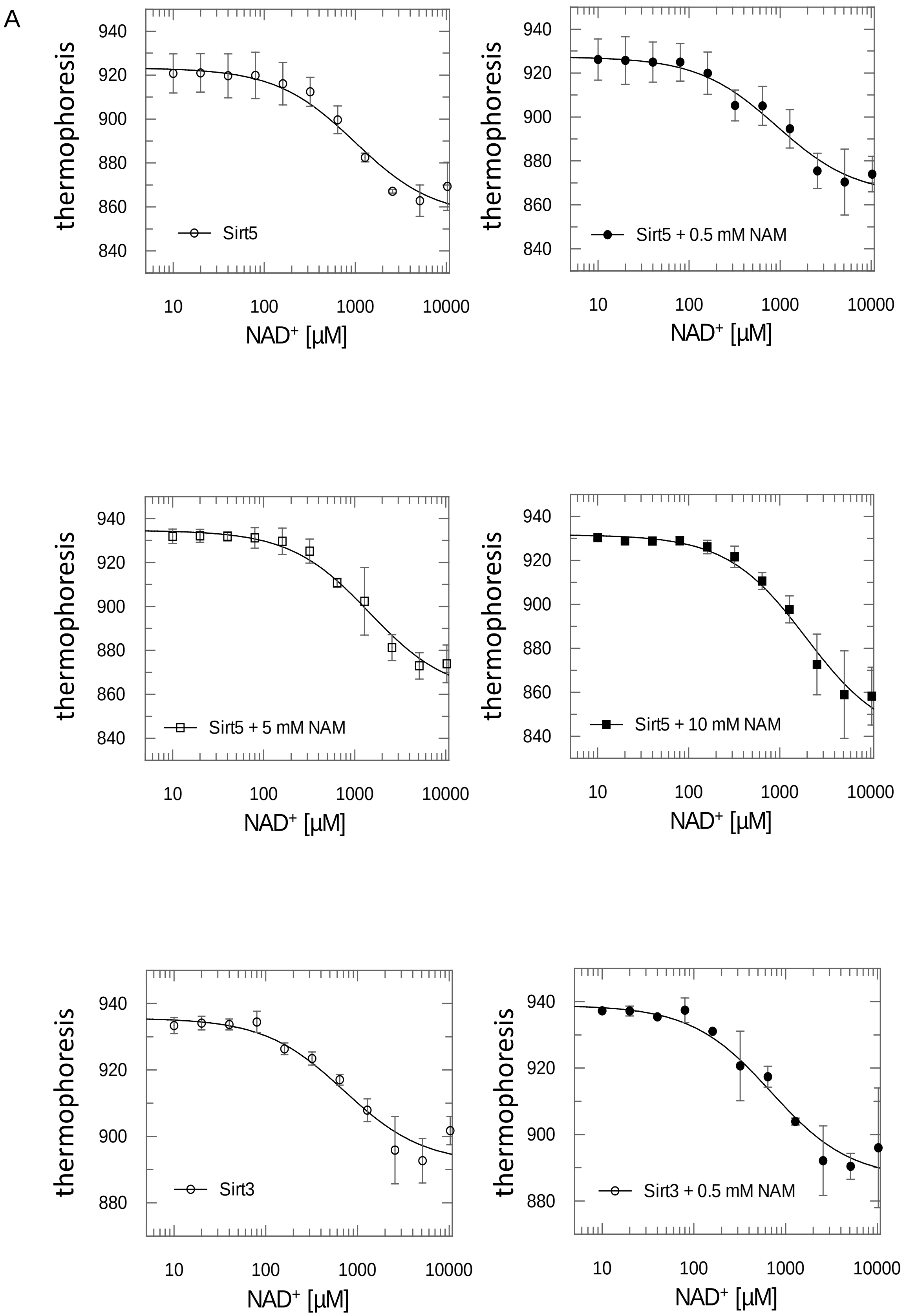

B

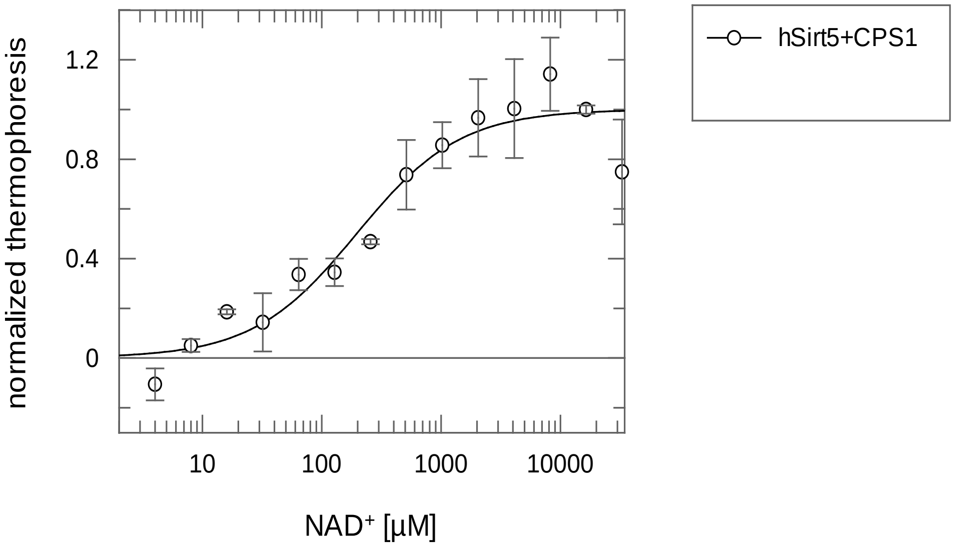
hSirt5+CPS1
normalized thermophoresis
1.2
0.8
0.4
0
10
100
1000
10000
NAD⁺ [µM]

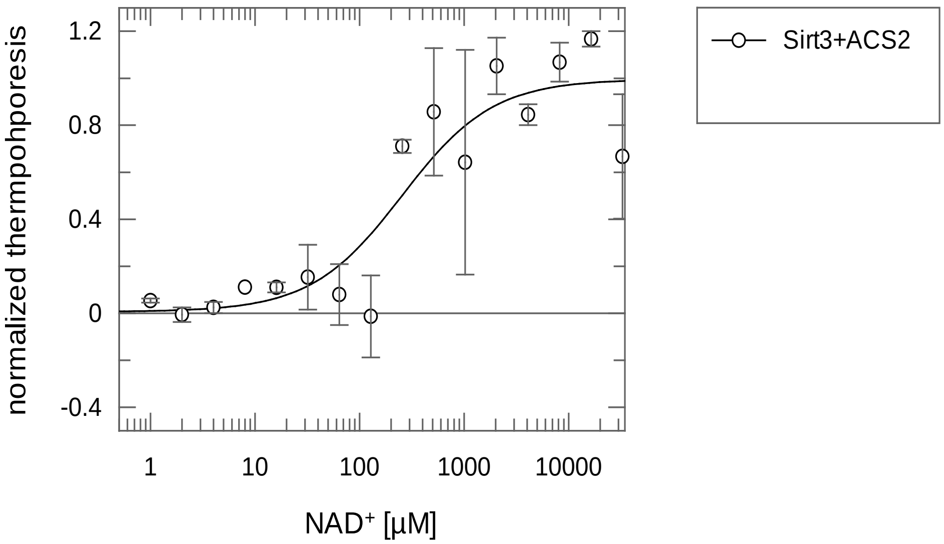
Sirt3+ACS2
normalized thermpohporesis
1.2
0.8
0.4
0
-0.4
1
10
100
1000
10000
NAD⁺ [µM]
