# Supplementary material for: Sirt5 Deacylation Activities Show Differential Sensitivities to Nicotinamide Inhibition
Source: PLoS One. 2012 Sep 19;7(9):e45098. doi: 10.1371/journal.pone.0045098 (PMC3446968; doi:10.1371/journal.pone.0045098)

Suppl. Fig. S3: Nicotinamide (NAM) dose response curves for various Sirtuins. The same set of data for human Sirt3 and Sirt5 from Fig. 2A is included in all graphs for comparison.

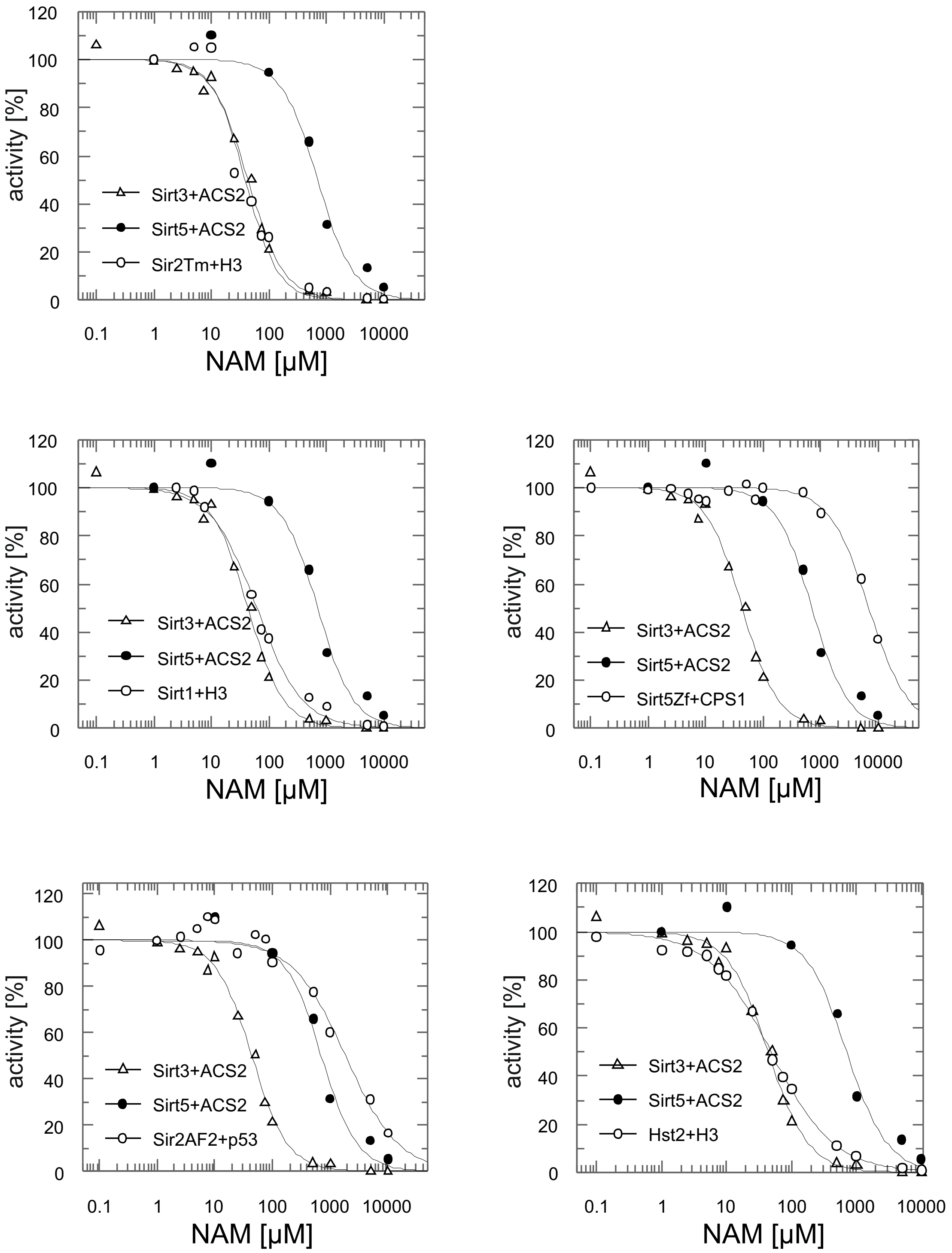

Supplement: Figure S3 — Nicotinamide (NAM) dose response curves for various Sirtuins. (PDF) [file pone.0045098.s003.pdf]
