# Supplementary material for: Sirt5 Deacylation Activities Show Differential Sensitivities to Nicotinamide Inhibition
Source: PLoS One. 2012 Sep 19;7(9):e45098. doi: 10.1371/journal.pone.0045098 (PMC3446968; doi:10.1371/journal.pone.0045098)

Suppl. Fig. S4: (A) Sir2Af2 dependent deacylation of CPS1-Lys527 and nicotinamide (NAM) dose response curves for Sir2Af2-dependent deacylation of (B) CPS1-Lys527 and (C) Prx1-Lys197 (Data for Sir2Af2 wt + Prx1/Prx1succ from Fig. 5C/D are shown for comparison).

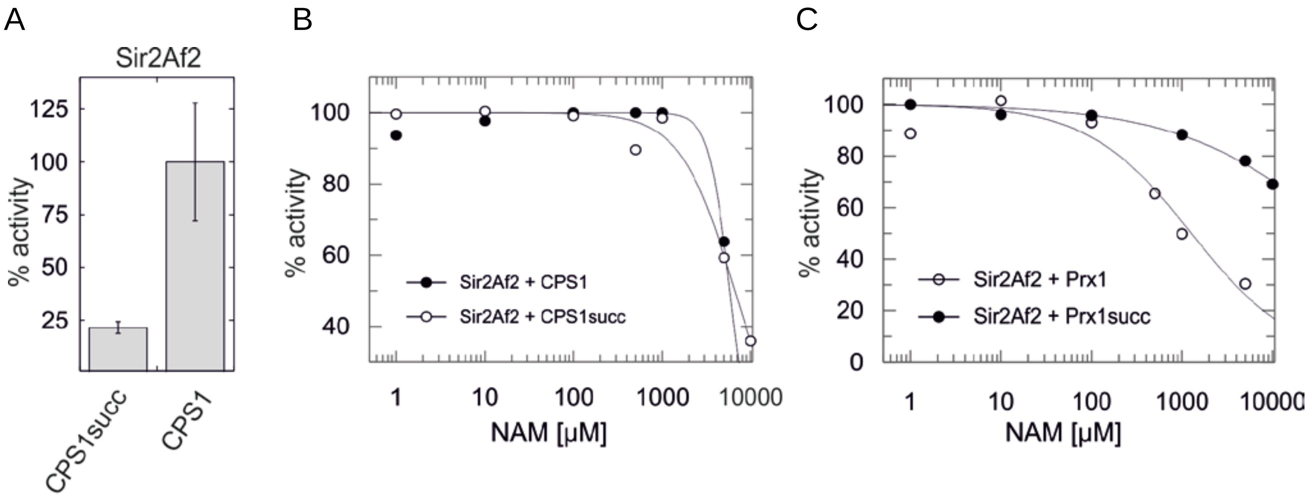

Supplement: Figure S4 — (PDF) [file pone.0045098.s004.pdf]
