# Supplementary material for: Sirt5 Deacylation Activities Show Differential Sensitivities to Nicotinamide Inhibition
Source: PLoS One. 2012 Sep 19;7(9):e45098. doi: 10.1371/journal.pone.0045098 (PMC3446968; doi:10.1371/journal.pone.0045098)
Supplement: Methods S1 — (PDF) [file pone.0045098.s005.pdf]

Supplementary Methods

Recombinant expression and purification of human Sirt1, Sirt3, and Sirt5, zebrafish Sirt5, yeast Hst2, *Archeoglobus fulgidus* Sir2Af2, and *Thermotoga maritima* Sir2Tm. The gene fragments of human Sirt1 (full length), human Sirt3 (residues 114-380), human Sirt5 (residues 34-302), zebrafish Sirt5 (residues 30-298), and yeast Hst2 (full length) were cloned into pET15b (Sirt1; Novagen, Darmstadt, Germany) or pET151/D-Topo (Invitrogen, Carlsbad, USA), resulting in an N-terminal 6x His tag and a TEV protease cleavage site. Proteins were expressed in *E. coli* BL21DE3 Rosetta 2 (Sirt1, Sirt3 and Sirt5, zebrafish Sirt5) or *E. coli* BL21DE3 (Hst2), respectively. Cells were cultured at 37 °C to an OD_{600} of 0.6, expression induced by adding 0.5 mM isopropylthiogalactopyranoside (IPTG), and culturing continued at 20 °C over night. Cells were harvested by centrifugation, resuspended in lysis buffer (*Sirt1*: 50 mM Tris/HCl pH 7.0, 300 mM NaCl; *Sirt3, Sirt5, zebrafish Sirt5*: 50 mM Tris/HCl pH 7.8, 200 mM NaCl; *Hst2*: 20 mM Tris/HCl pH 8.5, 500 mM NaCl) supplemented with 0.5 mM phenylmethylsulfonylfluorid (PMSF) and protease inhibitor mix (Serva, Heidelberg, Germany) and disrupted using a Microfluidizer (Microfluidics, Newton, USA). Cell lysates were cleared by centrifugation and supplemented with Talon beads (Clontech, Mountain View, USA) and 10 mM imidazole. Sirt1 was in addition incubated with 20 µg/ml RNAse, 10 µg/ml DNAse in 20 mM Tris/HCl pH 7.5, 25 mM NaCl, 0.2 mM PMSF, 5 mM $MgCl_2$ for 30 min at 4 °C. Beads were washed with buffer A (*Sirt1*: 50 mM Tris/HCl pH 7.0, 300 mM NaCl, 10 mM imidazole, 0.2 mM PMSF, protease inhibitor mix; *Sirt3, Sirt5, zebrafish Sirt5*: 50 mM Tris/HCl pH 7.8, 500 mM NaCl; *Hst2*: 20 mM Tris/HCl pH 8.5, 500 mM NaCl) and buffer B (*Sirt3, Sirt5, zebrafish Sirt5*: 50 mM Tris/HCl pH 7.8, 200 mM NaCl, 5 mM imidazole; *Hst2*: 20 mM Tris/HCl pH 8.5, 150 mM NaCl, 20 mM imidazole), and the proteins eluted in elution buffer (Sirt1: 50 mM Tris/HCl pH 7.0, 300 mM NaCl, 200 mM imidazole; Sirt3, *Sirt5, zebrafish Sirt5*: 50 mM Tris/HCl pH 7.8, 200 mM NaCl, 250 mM

imidazole; *Hst2*: 20 mM Tris/HCl pH 8.5, 150 mM NaCl, 150 mM imidazole). The proteins were then subjected to gelfiltration using a Superdex 200 column (Sirt1) or a Superose12 column (all other proteins) (GE Healthcare, Waukesha, USA) in gelfiltration buffer (*Sirt1*: 25 mM Hepes pH 7.5, 100 mM KCl, 2 mM DTT, 0.5 mM EDTA, 0.1 mM PMSF; *Sirt3, Sirt5, zebrafish Sirt5*: 20 mM Tris/HCl pH 7.8, 150 mM NaCl; *Hst2*: 20 mM Tris/HCl pH 8.5, 100 mM NaCl, 10 mM DTT). Sirt1 was further purified by ion exchange chromatography using a HiTrap Q HP column (GE Healthcare, Waukesha, USA) and a linear gradient of buffer A (25 mM Hepes pH 7.5, 100 mM KCl, 2 mM DTT, 0.5 mM EDTA, 0.1 mM PMSF) and buffer B (buffer A with KCl increased to 500 mM).
